# A novel circular RNA hsa_circRNA_103809/miR-377-3p/GOT1 pathway regulates cisplatin-resistance in non-small cell lung cancer (NSCLC)

**DOI:** 10.1186/s12885-020-07680-w

**Published:** 2020-12-04

**Authors:** Xiang Zhu, Jing Han, Huiyin Lan, Qingren Lin, Yuezhen Wang, Xiaojiang Sun

**Affiliations:** The Cancer Hospital of the University of Chinese Academy of Sciences (Zhejiang Cancer Hospital), Institute of Basic Medicine and Cancer (IBMC), Chinese Academy of Sciences, Hangzhou, 310022 Zhejiang China

**Keywords:** Non-small cell lung cancer, hsa_circRNA_103809, miR-377-3p, Glutamate oxaloacetate transaminase 1, Cisplatin-resistance

## Abstract

**Background:**

Cisplatin is the first-line chemotherapeutic drug for non-small cell lung cancer (NSCLC), and emerging evidences suggests that targeting circular RNAs (circRNAs) is an effective strategy to increase cisplatin-sensitivity in NSCLC, but the detailed mechanisms are still not fully delineated.

**Methods:**

Cell proliferation, viability and apoptosis were examined by using the cell counting kit-8 (CCK-8) assay, trypan blue staining assay and Annexin V-FITC/PI double staining assay, respectively. The expression levels of cancer associated genes were measured by using the Real-Time qPCR and Western Blot analysis at transcriptional and translated levels. Dual-luciferase reporter gene system assay was conducted to validated the targeting sites among hsa_circRNA_103809, miR-377-3p and 3′ untranslated region (3’UTR) of GOT1 mRNA. The expression status, including expression levels and localization, were determined by immunohistochemistry (IHC) assay in mice tumor tissues.

**Results:**

Here we identified a novel hsa_circRNA_103809/miR-377-3p/GOT1 signaling cascade which contributes to cisplatin-resistance in NSCLC in vitro and in vivo. Mechanistically, parental cisplatin-sensitive NSCLC (CS-NSCLC) cells were subjected to continuous low-dose cisplatin treatment to generate cisplatin-resistant NSCLC (CR-NSCLC) cells, and we found that hsa_circRNA_103809 and GOT1 were upregulated, while miR-377-3p was downregulated in CR-NSCLC cells but not in CS-NSCLC cells. In addition, hsa_circRNA_103809 sponged miR-337-3p to upregulate GOT1 in CS-NSCLC cells, and knock-down of hsa_circRNA_103809 enhanced the inhibiting effects of cisplatin on cell proliferation and viability, and induced cell apoptosis in CR-NSCLC cells, which were reversed by downregulating miR-377-3p and overexpressing GOT1. Consistently, overexpression of hsa_circRNA_103809 increased cisplatin-resistance in CS-NSCLC cells by regulating the miR-377-3p/GOT1 axis. Finally, silencing of hsa_circRNA_103809 aggravated the inhibiting effects of cisplatin treatment on NSCLC cell growth in vivo.

**Conclusions:**

Analysis of data suggested that targeting the hsa_circRNA_103809/miR-377-3p/GOT1 pathway increased susceptibility of CR-NSCLC cells to cisplatin, and this study provided novel targets to improve the therapeutic efficacy of cisplatin for NSCLC treatment in clinic.

**Supplementary Information:**

The online version contains supplementary material available at 10.1186/s12885-020-07680-w.

## Background

Non-small cell lung cancer (NSCLC) is a common malignancy with high morbidity and mortality, which has increased in incidence in the last decades [1, 2]. According to the report in 2015, the crude and age-adjusted incidences of NSCLC in China are 54.20 per 100,000 people [3], and the 5-year rate for patients with metastatic NSCLC is less than 5% [1, 2]. Currently, The efficacy of the current therapeutic strategies, which include surgical resection [4, 5], chemotherapy [6], radiotherapy [7, 8], and immunotherapy [9, 10], are limited by advance- stage disease, chemo-resistance and radio-resistance [11, 12]. The chemotherapeutic drug cisplatin is currently used as first-line treatment for NSCLC [13, 14]. Recent evidence show that continuous long-term stimulation of NSCLC cells by cisplatin caused alteration of multiple cancer associated Circular RNAs (circRNAs), resulting in a decrease in the effectiveness of the drug [13, 14]. Uncovering the underlying mechanisms leading to this resistance might solve this problem. Based on the above information, by searching the online Pubmed database (https://pubmed.ncbi.nlm.nih.gov/), we selected hsa_circRNA_103809 for further investigations in this study, and the main reason is that hsa_circRNA_103809 acted as an oncogene to promote cancer development in colorectal cancer [15], breast cancer [16], hepatocellular carcinoma [17], gastric cancer [18] and lung cancer [19]. However, to date, the role of hsa_circRNA_103809 in drug resistance in cancer remains largely unknown and therefore important to investigate.

According to recent advances in circRNAs research, circRNAs exert their biological functions through serving as RNA sponges to competitively bind to microRNAs (miRNAs) and regulating gene expression [20–22]. Taking hsa_circRNA_103809 as an example, previous publications indicate that hsa_circRNA_103809 sponged multiple miRNAs, including miR-101-3p [18], miR-532-3p [15], miR-4302 [19], miR-620 [23] and miR-377-3p [17]. According to our preliminary work (data not shown), continuous low-dose cisplatin treatment specifically and preferentially decreased the expression levels miR-377-3p in cisplatin-resistant NSCLC (CR-NSCLC) cells. Consistently, Ling Liu et al. reported that miR-377-3p was downregulated in cisplatin-resistant osteosarcoma cells and tissues [24], which enlightened us to choose miR-377-3p for further investigations. In addition, miR-377-3p functioned as a tumor suppressor to hamper the development of multiple cancers, such as breast cancer [25], gastric cancer [26], ovarian cancer [27] and NSCLC [28–30]. Interestingly, miR-377-3p regulated drug resistance in cancer treatment, and Ling Liu et al. reported that miR-377-3p participated in the regulation of cisplatin-resistance in osteosarcoma [24], but the role of miR-377-3p in regulating cisplatin-sensitivity in NSCLC is still unknown.

Glutamate oxaloacetate transaminase 1 (GOT1) mainly regulates cellular glutaminolysis, which converts glutamate (Glu) into a-ketoglutaric acid (a-KG) and is crucial for sustaining cancer progression [31, 32]. Inhibition of GOT1 had been validated to be an effective strategy to impair cancer growth in pancreatic cancer [32] and lung cancer [31]. Notably, previous data suggests that cisplatin regulated mitochondrial GOT1 to induce nephrotoxicity in rats [33], which rendered the possibility that targeting GOT1 might help to increase the therapeutic efficacy of cisplatin in NSCLC. In addition, recent studies have suggested that miRNAs could bind to the 3′ untranslated regions (3’UTRs) of GOT1 mRNA, resulting in GOT1 degradation and downregulation [34, 35], and Zhang K et al. found that miR-9 targeted GOT1 to regulate cell ferroptosis in melanoma [34]. By conducting the online miRDB software (http://mirdb.org/), we predicted that miR-377-3p potentially bound to the 3’UTR of GOT1 mRNA. Given the fact that hsa_circRNA_103809 sponged miR-377-3p in NSCLC cells, we speculated that hsa_circRNA_103809 might regulate GOT1 through miR-377-3p in a competing endogenous RNA (ceRNA)-dependent manner.

Based on the published literatures, by conducting in vitro and in vivo experiments, this study identified that the hsa_circRNA_103809/miR-377-3p/GOT1 pathway regulated cisplatin-resistance in NSCLC cells, and targeting this pathway improved cisplatin-sensitivity in NSCLC, which provided potential avenues for improving NSCLC treatment in the clinic.

## Methods

### Cell culture and induction of cisplatin-resistant NSCLC (CR-NSCLC) cells

The parental CS-NSCLC cell lines, including A549 (ATCC® CCL-185™), H1299 (ATCC® CRL-5803™) and Calu-3 (ATCC® HTB-55™), were purchased from American Type Culture Collection (ATCC, USA) in Jan. 2019, and cultured in the incubator with standard culture conditions (37 °C and 5% CO_2_ humidified atmosphere). The cells were authenticated by STR profiling and were identified as mycoplasma-free by a commercial third-party company (Abace Biotechnology, Beijing, China). The Roswell Park Memorial Institute 1640 medium (RPMI-1640, HyClone, USA) containing 10% fetal bovine serum (FBS, Gibco, USA) was used for cell cultivation. According to the experimental procedures provided by the previous work [36, 37] and our preliminary experiments (data not shown), the CS-NSCLC cells were exposed to continuous low-dose cisplatin treatment, ranged from 0.5 μg/ml to 5 μg/ml, for 80 days in a step-wise manner to generate descendent CR-NSCLC cells (A549/DDP, H1299/DDP and Calu-3/DDP). After that, the CR-NSCLC cells were stimulated with high-dose cisplatin (25 μg/ml) for 0 h, 24 h, 48 h and 72 h, to validate the successful induction of CR-NSCLC cells.

### Vectors transfection

The overexpression and downregulation vectors for hsa_circRNA_103809 and GOT1, and miR-377-3p mimic and inhibitor were designed and synthesized by Sangon Biotech (Shanghai, China), and the above vectors were delivered into CS-NSCLC and CR-NSCLC cells to manipulate genes expressions by using the commercial Lipofectamine 2000 reagent (Invitrogen, USA), based on the experimental protocols provided by the producer. After that, Real-Time qPCR was conducted to validate the transfection efficiency of the above vectors. The sequence of siRNA for hsa_circRNA_103809 (5′- CAG TCT TAT CTC ACT TTA CTG GAT A-3′); The primers for hsa_circRNA_103809 overexpression plasmid construction (Forward: 5′-TAA TAA CTA AGA TCT GGT ACC GTT TTG ATG ATG AAA CAG AAG ATC AGC-3′, Reverse: 5′-GAA GCA TGA ATT CAA GGT ACC CAC CAA GTC TTC ACA ACT CCT GTC-3′); miR-377-3p mimic (5′-AUC ACA CAA AGG CAA CUU UUG U-3′) and inhibitor (5′-ACA AAA GUU GCC UUU GUG UGA U-3′); The short hairpin RNA (shRNA) for GOT1 downregulation (5′- CCG GGC GTT GGT ACA ATG GAA CAA ACT CGA GTT TGT TCC ATT GTA CCA ACG CTT TTT G-3′); The primers used for GOT1 overexpression (5′-CAA CTG GGA TTG ACC CAA CT-3′, Reverse: 5′-GGA ACA GAA ACC GGT GCT T-3′).

### Cell counting kit-8 (CCK-8) assay

The NSCLC cells were pre-transfected with the above vectors, cultured in 96-well plates at standard culture conditions, and were subjected to cisplatin (25 μg/ml) stimulation for 0 h, 24 h, 48 h and 72 h, respectively. After that, the CCK-8 reaction solution (AbMole, USA) was incubated with the cells in the volume of 20 μl per well for 4 h at the incubator. Then, the plates were vortexed to thoroughly mix the cells with the solution, and were placed in a microplate reader (ThermoFisher Scientific, USA) to measure the optical density (OD) values at the wavelength of 450 nm, which could be used to represent relative cell proliferation in the cells.

### Trypan blue staining assay

The CR-NSCLC and CS-NSCLC cells were pre-transfected with different vectors, and stimulated by using the cisplatin. Then, the cells were prepared and stained with trypan blue staining solution obtained from Invitrogen (USA) for 20 min at room temperature. After that, a light microscope was used to observe and count the number for dead blue cells, which were used to evaluate cell viability according to the following formula: Cell viability (%) = (Total cells – Dead blue cells)/Total cells * 100%.

### Annexin V-FITC/PI double staining assay

A apoptosis detection kit (Invitrogen, USA) was used to examine cell apoptosis in CS-NSCLC cells and CR-NSCLC cells, based on the protocols provided by the manufacturer. In brief, the cells were harvested and prepared, and subsequently stained with Annexin V-FITC and propidium iodide (PI) for 25 min at room temperature without light exposure. After that, a flow cytometer (FCM, ThermoFisher Scientific, USA) was used to examine the cell death ratio in NSCLC cells. Specifically, the early apoptotic cells were stained with Annexin V-FITC alone, the late apoptotic cells were stained with Annexin V-FITC and PI, and the necroptotic cells were stained with PI alone.

### Real-time qPCR

The NSCLC cells were subjected to differential treatments, and the TRIzol reagent (Invitrogen, USA) was employed to extract the total RNA. Specifically, 5 × 10 ^6^ cells were treated with 1 ml TRIzol solution for 5 min, and were subsequently treated with chloroform for 15 min at room temperature. Next, the upper water phase was collected, and was treated with 0.5 ml isopropyl alcohol for 10 min. After centrifugation with 12,000 g for 10 min, 75% ethyl alcohol was used to isolate and purify the total RNA. Next, the Real-Time qPCR was conducted to determine the expression levels of hsa_circRNA_103809, miR-377-3p and GOT1 mRNA, and the experimental procedures had all been documented in the previous publications [36, 37]. Of note, to detect hsa_circRNA_103809 levels, the total RNA must be pre-treated with RNase R enzyme (3 U/μg) for 20 min at 37 °C to eliminate linear RNA. The primer sequences for the involved genes are as follows: hsa_circRNA_103809 (Forward: 5′-ACG CAT TCT TCG AGA CCT CT-3′, Reverse: 5′-TGC CTG TAA CTC CTC TTC AGT-3′), miR-377-3p (Forward: 5′- ATC ACA CAA AGG CAA CTT TTG T-3′, Reverse: 5′- GGT GCA GGG TCC GAG GTA T-3′), GOT1 (Forward: 5′-TGC CAG TAG TGA AGA AAG TG-3′, Reverse: 5′-TAA GCG ATA GGA CCG AAT-3′), β-actin (Forward: 5′-GCT CGT CGT CGA CAA CGG CT-3′, Reverse: 5′-CAA ACA TGA TCT GGC TCA TCT TCT C-3′) and U6 (Forward: 5′-CTC GCT TCG GCA GCA CA-3′, Reverse: 5′-AAC GCT TCA CGA ATT TGC GT-3′).

### Western blot analysis

The RIPA lysis buffer was purchased from Solarbio (Beijing, China) to lyse the NSCLC cells/tissues and extract the total protein, according to the experimental procedures recorded in the previous publications [36, 37], the expression levels of GOT1, β-actin, cyclin D1, CDK2, cleaved caspase-3 and Bax were examined by using the Western Blot analysis. Specifically, the 40 μg/lane protein lysates were separated by using the 10 -15% SDS-PAGE, and the target protein bands were transferred onto the PVDF membranes (Millipore, USA). Next, the membranes were incubated with 5% skim milk for 70 min at room temperature for blocking, and the membranes were probed with the primary antibodies against GOT1 (1:1500, MW: 50 kDa, #PA5–24634, Thermo, USA), β-actin (1:2000, MW: 42 kDa, #ab6276, Abcam, UK), cyclin D1 (1:1500, MW: 35 kDa, #ab40754, Abcam, UK), CDK2 (1:2000, MW: 33 kDa, #ab32147, Abcam, UK), cleaved caspase-3 (1:1500, MW: 17 kDa, #ab32042, Abcam, UK) and Bax (1:1500, MW: 21 kDa, #ab32503, Abcam, UK) overnight at 4 °C. After washing by PBS buffer for 3 times, the PVDF membranes were incubated with the secondary antibody (Abcam, UK) for 120 min at room temperature. Finally, the protein bands were visualized by ECL system (GE Healthcare Bio-science, USA) and quantified by using the Image J software.

### Dual-luciferase reporter gene system assay

The binding sites of miR-377-3p with hsa_circRNA_103809 and 3′ UTR region of GOT1 mRNA were predicted by the online miRDB software (http://mirdb.org/), and validated by using the dual-luciferase reporter gene system, and the detailed experimental procedures had been well documented in the previous literatures [36, 37]. Briefly, the targeting sites in hsa_circRNA_103809 and GOT1 were mutated, and named as Mut-circRNA and Mut-GOT1, respectively. Correspondingly, the original wild-type (Wt) genes were named as Wt-CircRNA and Wt-GOT1. The above sequences were cloned into the luciferase reporter vectors by Sangon Biotech (Shanghai, China), and the schematic image for the luciferase reporters was shown in Figure S3. The above vectors were delivered into NSCLC cells co-transfected with miR-377-3p mimic and inhibitor for 48 h. After that, the commercial dual-luciferase reporter assay kit (Promega, USA) was used to measure relative luciferase activities in the cells.

### Xenograft tumor-bearing mice models

The CR-NSCLC cells were pre-transfected with different vectors, and were subcutaneously injected into the dorsal flank of male nude mice (*N* = 20), and the age of the mice ranged from 6 to 8 weeks. Each mouse was injected with 5 × 10^6^ cells, at 7 days post-injection, the tumor were subjected to high-dose cisplatin (10 μg/ml) treatment every 3 days for 2 weeks. The above mice were equally divided into 4 groups, including Control, Cisplatin, KD-circRNA and Cisplatin + KD-circRNA, each group had 5 mice. The mice were sacrificed at 35 days post-injection. After that, the mice tumor tissues were collected, and the expression levels of proliferation associated proteins (cyclin D1 and CDK2) and apoptosis associated proteins (cleaved caspase-3 and Bax) were examined by using Western Blot analysis, and the expressions/localization of Ki67 protein in mice tissues were determined by Immunohistochemistry (IHC). All the animal experiments were approved by the Ethics Committee of The Cancer Hospital of the University of Chinese Academy of Sciences (Zhejiang Cancer Hospital), Institute of Basic Medicine and Cancer (IBMC), and the approval number was 2020-12-002.

### Immunohistochemistry (IHC)

The mice tumor tissues were collected and spliced into sections of 5 μm thickness, and IHC assay was conducted to determine the expressions and localization of Ki67 protein in the mice tissues, the detailed experimental procedures can be found at the previous publications [36, 37]. The antibody against Ki67 protein was bought from Abcam (UK), and was diluted at the ratio of 1:400.

### Statistical analysis

Data analysis was conducted by using the SPSS 18.0 software, and the data was represented as Means ± Standard Deviation. The comparisons between two groups were performed by using the Student’s t-test, and the comparisons among multiple groups were conducted by using one-way ANOVA analysis. Each experiment was repeated at least 3 times, *P < 0.05 could be regarded as statistical significance.

## Results

### The expression patterns of hsa_circRNA_103809, miR-377-3p and GOT1 in CS-NSCLC and CR-NSCLC cells

**Fig. 1:**
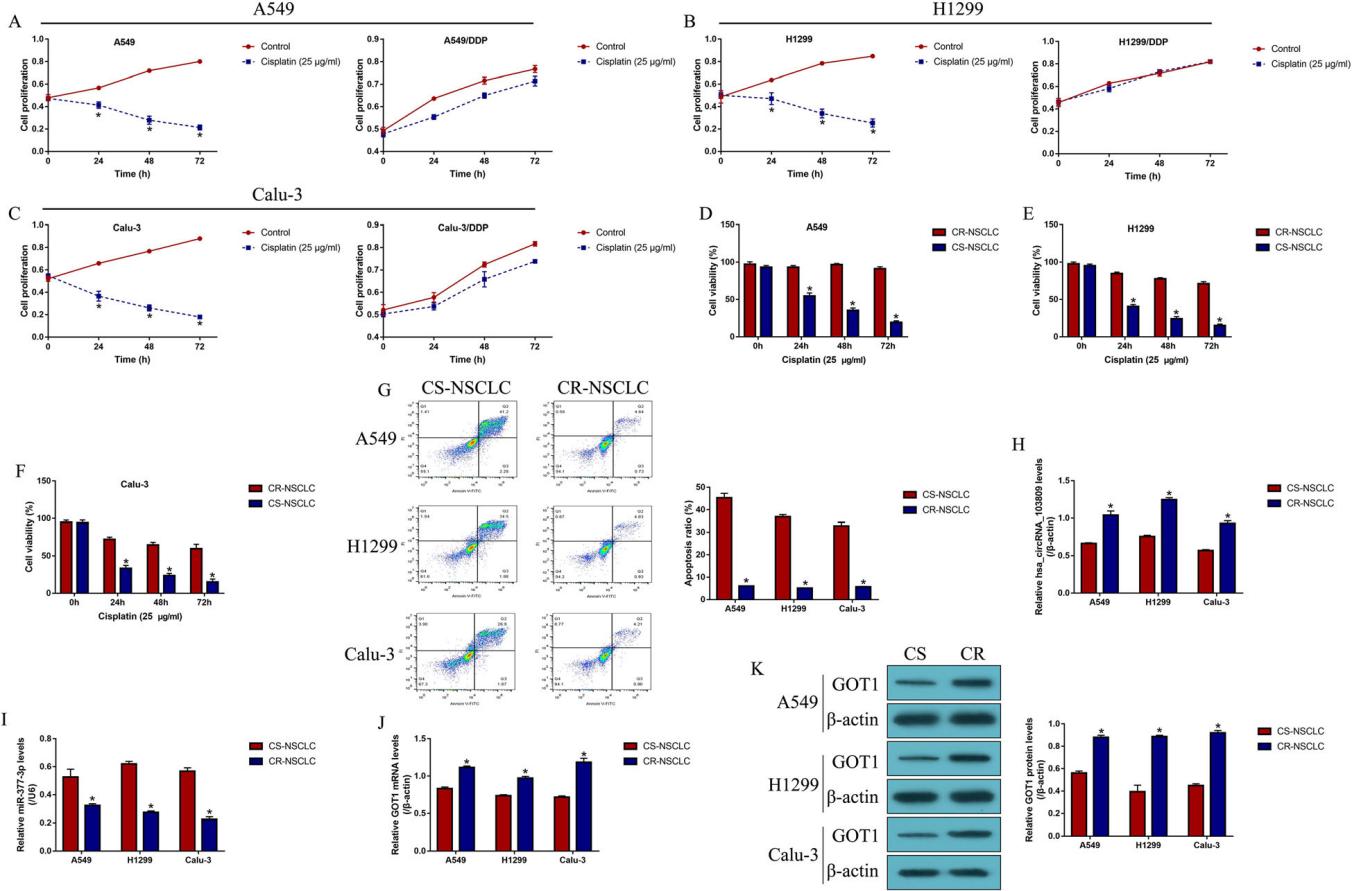
Continuous low-dose cisplatin pressure changed the expression patterns of hsa_circRNA_103809, miR-377-3p and GOT1 in NSCLC cells. The parental CS-NSCLC cells (A549, H1299 and Calu-3) were subjected to continuous low-dose cisplatin treatment to generate CR-NSCLC cells (A549/DDP, H1299/DDP and Calu-3/DDP). **a-c** Cell proliferation abilities in CS-NSCLC and CR-NSCLC cells were determined by using the CCK-8 assay (Note: “Control: without cisplatin stimulation”). **d-f** Trypan blue staining assay was conducted to evaluate NSCLC cell viability. **g** Cell apoptosis ratio was measured by using the Annexin V-FITC/PI double staining method. Real-Time qPCR was used to examine the expression levels of (**h**) hsa_circRNA_103809, **i** miR-377-3p and **j** GOT1 mRNA in NSCLC cells. **k** Western Blot analysis was employed to determine the protein levels of GOT1 in NSCLC cells, full-length blots/gels are presented in Supplementary Figure S4. Each experiment was repeated at least 3 times. *P < 0.05

The CS-NSCLC cell lines (A549, H1299 and Calu-3) were subjected to continuous low-dose cisplatin treatment to generate CR-NSCLC cells (A549/DDP, H1299/DDP and Calu-3/DDP), which simulated the realistic conditions of cisplatin-resistance in NSCLC patients in vitro. Next, the NSCLC cells were stimulated with high-dose cisplatin (25 μg/ml) for 0 h, 24 h, 48 h and 72 h, and cell proliferation was evaluated by the CCK-8 assay (Fig. 1a-c). The results showed that the proliferation abilities in CS-NSCLC cells but not in CR-NSCLC cells, were significantly inhibited by cisplatin treatment (Fold changes (72 h): 0.352 vs. 0.983 in A549 and A549/DDP cells; 0.261 vs. 1.212 in H1299 and H1299/DDP cells; 0.189 vs. 0.783 in Calu-3 and Calu-3/DDP cells. Figure 1a-c). Consistently, the trypan blue staining assay results validated that cisplatin significantly inhibited cell viability of CS-NSLCC cells compared to CR-NSCLC cells (Fold changes (72 h): 0.218 vs. 1.093 in A549 and A549/DDP cells; 0.328 vs. 0.996 in H1299 and H1299/DDP cells; 0.421 vs. 0.864 in Calu-3 and Calu-3/DDP cells. Figure 1d-f). Next, the cells were stained with Annexin V-FITC and PI, and cell apoptosis was detected by using flow cytometry (FCM) (Fig. 1g). As expected, the data suggested that cisplatin induced apoptotic cell death in CS-NSCLC cells, compared to the CR-NSCLC cells (Fold changes: 9.07, 8.56 and 6.38 vs. CR-NSCLC cells, Fig. 1g), suggesting that CR-NSCLC cells were much more resistant to cisplatin treatment. Next, the expression status of hsa_circRNA_103809, miR-377-3p and GOT1 were examined in the NSCLC cells, and we found that hsa_circRNA_103809 (Fig. 1h) and GOT1 (Fig. 1j, k) were upregulated, while miR-377-3p (Fig. 1i) was downregulated in CR-NSCLC cells, suggesting that continuous low-dose cisplatin pressure altered the expression status of hsa_circRNA_103809, miR-377-3p and GOT1 in CR-NSCLC cells.

### The regulatory mechanisms of hsa_circRNA_103809, miR-377-3p and GOT1 in NSCLC cells

**Fig. 2:**
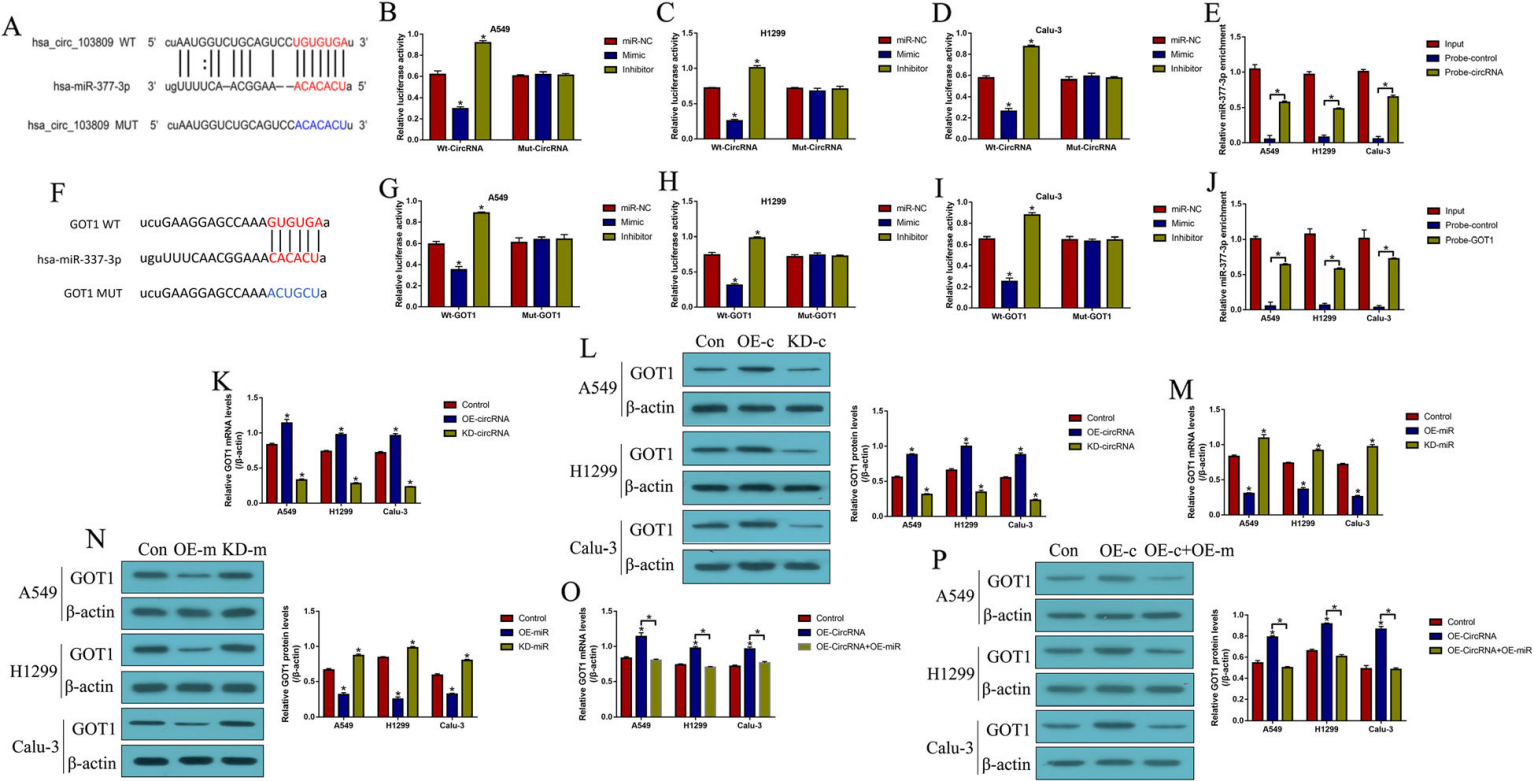
The regulatory link between hsa_circRNA_103809, miR-377-3p and GOT1. The online miRDB software (http://mirdb.org/) indicated that miR-377-3p potentially bound to (**a**) hsa_circRNA_103809 and (F) 3′ untranslated region (3’UTR) of GOT1 mRNA. Dual-luciferase reporter gene system assay was performed to validate the binding sites of miR-377-3p with (**b-d**) hsa_circRNA_103809 and (G-I) 3′ UTR region of GOT1 mRNA in NSCLC cells. RNA pull-down assay suggested that miR-377-3p tended to be enriched by the biotin-labelled probes for (**e**) hsa_circRNA_103809 and (**j**) GOT1 mRNA. (**k, l**) GOT1 was positively regulated by hsa_circRNA_103809 in NSCLC cells, full-length blots/gels are presented in Supplementary Figure S5A. (**m, n**) MiR-377-3p inhibited GOT1 expressions in NSCLC cells, full-length blots/gels are presented in Supplementary Figure S5B. (**o, p**) Hsa_circRNA_103809 sponged miR-377-3p to upregulate GOT1 in NSCLC cells, full-length blots/gels are presented in Supplementary Figure S5C. (Note: “Control: without vectors transfection”). Each experiment was repeated at least 3 times. *P < 0.05

**Fig. 3:**
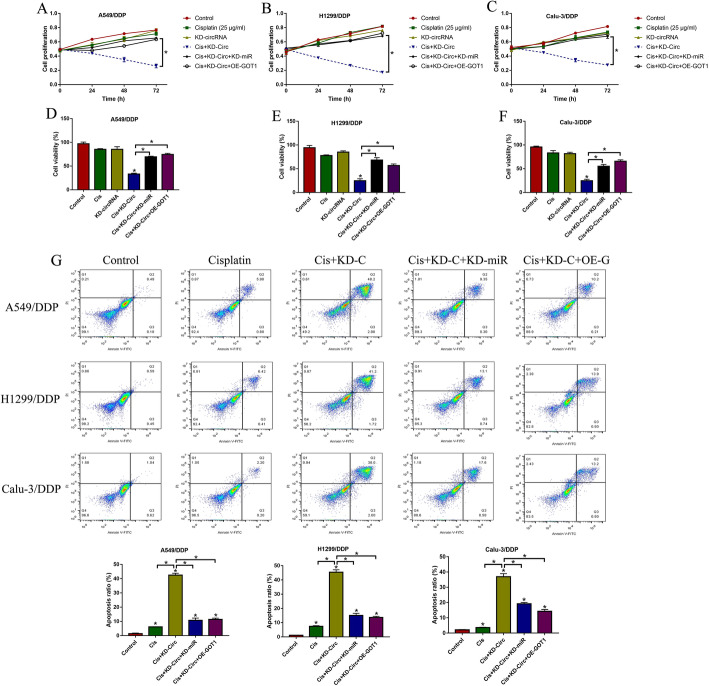
Knock-down of hsa_circRNA_103809 increased cisplatin-sensitivity in CR-NSCLC cells by targeting miR-377-3p and GOT1. **a-c** CCK-8 assay was performed to determine cell proliferation abilities. **d-f** Trypan blue staining assay was used to examine cell viability. **g** Annexin V-FITC/PI double staining assay was performed to measure cell apoptosis ratio in CR-NSCLC cells. (Note: “Control: without vectors transfection and cisplatin treatment”). Each experiment was repeated at least 3 times. *P < 0.05

By using the online miRDB software (http://mirdb.org/), we predicted a relationship among hsa_circRNA_103809, miR-377-3p and GOT1 (Fig. 2). Mechanistically, binding sites for miR-377-3p with hsa_circRNA_103809 (Fig. 2a) and the 3′ untranslated region (3’UTR) of GOT1 mRNA (Fig. 2f) were predicted, which were validated by the subsequent dual-luciferase reporter gene system. The results showed that miR-377-3p mimic targeted the binding sites in hsa_circRNA_103809 (Fig. 2b-d) and 3’UTR of GOT1 mRNA (Fig. 2g-i) to decrease the relative luciferase activities in CS-NSCLC cells, while miR-377-3p inhibitor had the opposite effects (Fig. 2b-d, g-i). Additionally, the RNA pull-down assay verified that miR-337-3p could be enriched by biotin-labelled hsa_circRNA_103809 (Fig. 2e) and GOT1 mRNA (Fig. 2j) probes but not in the control probes. Next, the overexpression and downregulation vectors for hsa_circRNA_103809 were transfected into CS-NSCLC cells (Figure S2A), and the results showed that hsa_circRNA_103809 positively regulated GOT1 expressions in CS-NSCLC cells (Fig. 2k, l). In addition, the miR-337-3p mimic and inhibitor were delivered into CS-NSCLC cells (Figure S2B), and the results showed that miR-337-3p inhibited GOT1 expressions in CS-NSCLC cells (Fig. 2m, n). Of note, the promoting effects of hsa_circRNA_103809 overexpression on GOT1 were abrogated by upregulating miR-337-3p (Fig. 2o, p). The above results indicated that hsa_circRNA_103809 sponged miR-337-3p to upregulate GOT1 in CS-NSCLC cells.

### Knock-down of hsa_circRNA_103809 sensitized CR-NSCLC cells to cisplatin by regulating the miR-377-3p/GOT1 axis

Further experiments were conducted to investigate the regulating effects of hsa_circRNA_103809 on cisplatin-resistance in NSCLC. To achieve this, the silencing vectors for hsa_circRNA_103809 were transfected into CR-NSCLC cells to knock down hsa_circRNA_103809 (Figure S1A), and the CCK-8 results showed that either hsa_circRNA_103809 ablation or cisplatin treatment alone had little effects on cell proliferation abilities in CR-NSCLC cells, while silencing of hsa_circRNA_103809 enhanced the inhibiting effects of cisplatin on cell growth (Fig. 3a-c). Then, the miR-337-3p downregulation (Figure S1B) and GOT1 overexpression (Figure S1C) vectors were transfected into CR-NSCLC cells, and we found that the aggravating effects of hsa_circRNA_103809 ablation on cell proliferation in CR-NSCLC cells were abrogated by downregulating miR-337-3p and upregulating GOT1 (Fold changes (72 h): Cis + KD-circ+KD-miR and Cis + KD-circ+OE-GOT1 vs. Cis + KD-circ, 2.63 and 3.01 in A549/DDP; 4.32 and 4.18 in H1299/DDP cells; 2.31 and 2.19 in Calu-3/DDP. Figure 3a-c). In addition, the trypan blue staining assay results validated that knock-down of hsa_circRNA_103809 inhibited cell viability in CR-NSCLC cells through targeting miR-337-3p and GOT1 (Fold changes: Cis + KD-circ+KD-miR and Cis + KD-circ+OE-GOT1 vs. Cis + KD-circ, 2.12 and 1.98 in A549/DDP; 2.64 and 1.67 in H1299/DDP; 1.93 and 2.03 in Calu-3/DDP. Figure 3d-f). Consistently, as shown in Fig. 3g, downregulated hsa_circRNA_103809 aggravated the promoting effects of cisplatin-induced cell apoptosis in CR-NSCLC cells, which were also reversed by knocking down miR-337-3p and overexpressing GOT1 (Fold changes: Cis + KD-circ+KD-miR and Cis + KD-circ+OE-GOT1 vs. Cis + KD-circ, 0.19 and 0.21 in A549/DDP; 0.32 and 0.33 in H1299/DDP; 0.46 and 0.35 in Calu-3/DDP. Figure 3g).

### Upregulation of hsa_circRNA_103809 increased cisplatin-resistance in CS-NSCLC cells through miR-377-3p and GOT1

**Fig. 4:**
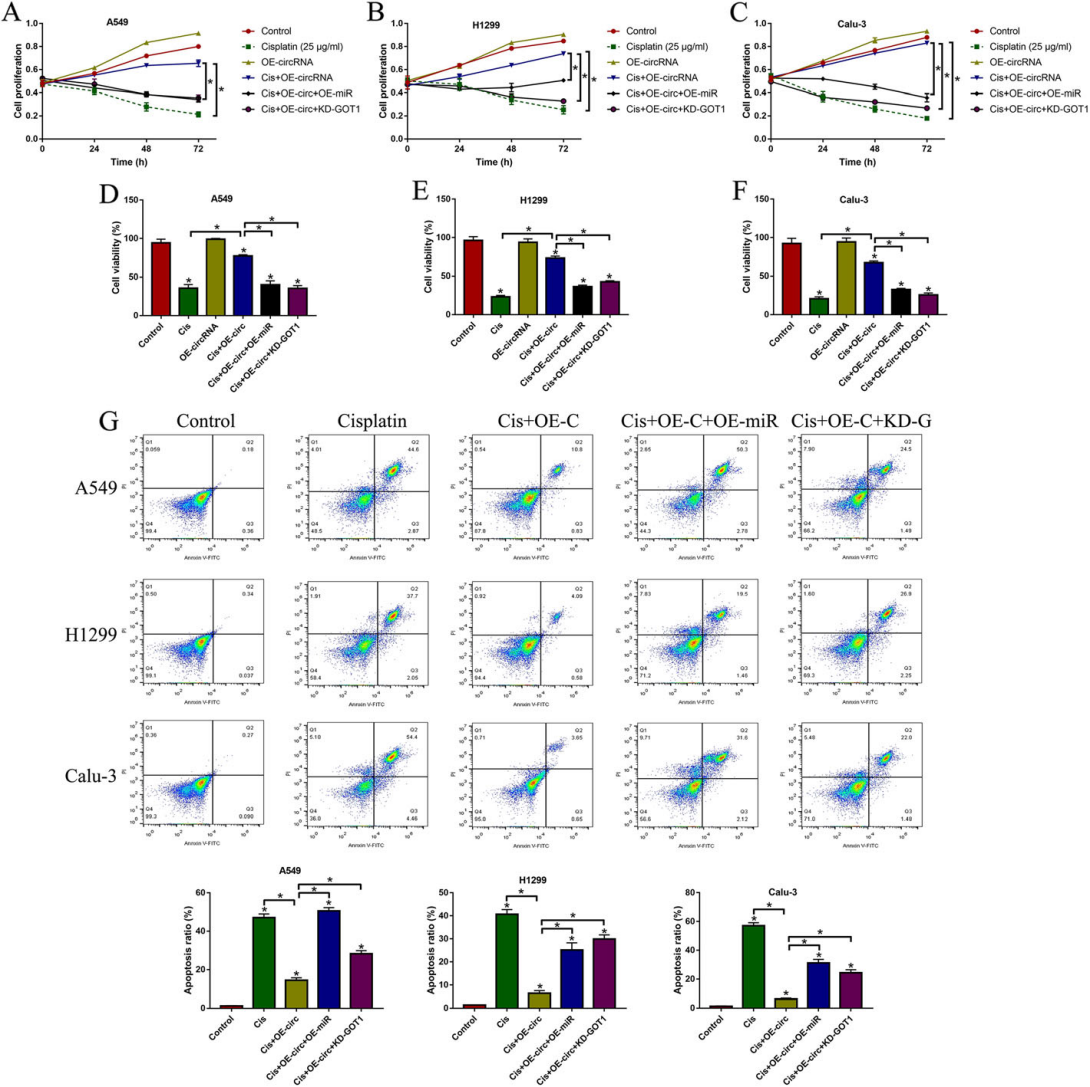
Upregulation of hsa_circRNA_103809 promoted cisplatin-resistance in CS-NSCLC cells. **a-c** Cell proliferation abilities were examined by using the CCK-8 assay. **d-f** Cell viability was evaluated by performing the trypan blue staining assay. **g** Cell apoptosis was determined by using the Annexin V-FITC/PI double staining assay. (Note: “Control: without vectors transfection and cisplatin treatment”). Each experiment was repeated at least 3 times. *P < 0.05

Next, we further confirmed that overexpression of hsa_circRNA_103809 regulated the miR-377-3p/GOT1 axis to increase cisplatin-resistance in the parental CS-NSCLC cells (A549, H1299 and Calu-3) (Fig. 4). Mechanistically, the hsa_circRNA_103809 overexpression vectors (Figure S2A), miR-377-3p mimic (Figure S2B) and GOT1 silencing vectors (Figure S2C) were delivered into the CS-NSCLC cells. Then, the cells were subjected to high-dose cisplatin (25 μg/ml) stimulation for 0 h, 24 h, 48 h and 72 h. The CCK-8 assay results indicated that upregulation of hsa_circRNA_103809 improved cell proliferation abilities in cisplatin-treated CS-NSCLC cells, which were abrogated by upregulating miR-337-3p and downregulating GOT1 (Fold changes (72 h): Cis + OE-circ+OE-miR and Cis + OE-circ+KD-GOT1 vs. Cis + OE-circ, 0.34 and 0.21 in A549; 0.21 and 0.26 in H1299; 0.43 and 0.32 in Calu-3. Figure 4a-c). Similarly, the trypan blue staining assay results also supported that hsa_circRNA_103809 overexpression rescued cell viability in CS-NSCLC cells under cisplatin treatment by sponging miR-337-3p and upregulating GOT1 (Fold changes: Cis + OE-circ+OE-miR and Cis + OE-circ+KD-GOT1 vs. Cis + OE-circ, 0.63 and 0.54 in A549; 0.54 and 0.61 in H1299; 0.32 and 0.28 in Calu-3. Figure 4d-f). Furthermore, we conducted Annexin V-FITC/PI double staining assay to determine cell apoptosis, and the results indicated that cisplatin significantly increased cell apoptosis ratio in CS-NSCLC cells, which were reversed by overexpressing hsa_circRNA_103809 (Fold changes: Cis + OE-circ+OE-miR and Cis + OE-circ+KD-GOT1 vs. Cis + OE-circ, 4.66 and 2.27 in A549; 4.76 and 6.58 in H1299; 8.66 and 6.03 in Calu-3. Figure 4g). Also, the inhibiting effects of hsa_circRNA_103809 overexpression on cell apoptosis in cisplatin-treated CS-NSCLC cells were abrogated by downregulating miR-337-3p and upregulating GOT1 (Fig. 4g).

### Targeting hsa_circRNA_103809 enhanced the inhibiting effects of cisplatin on CR-NSCLC cell growth in vivo

**Fig. 5:**
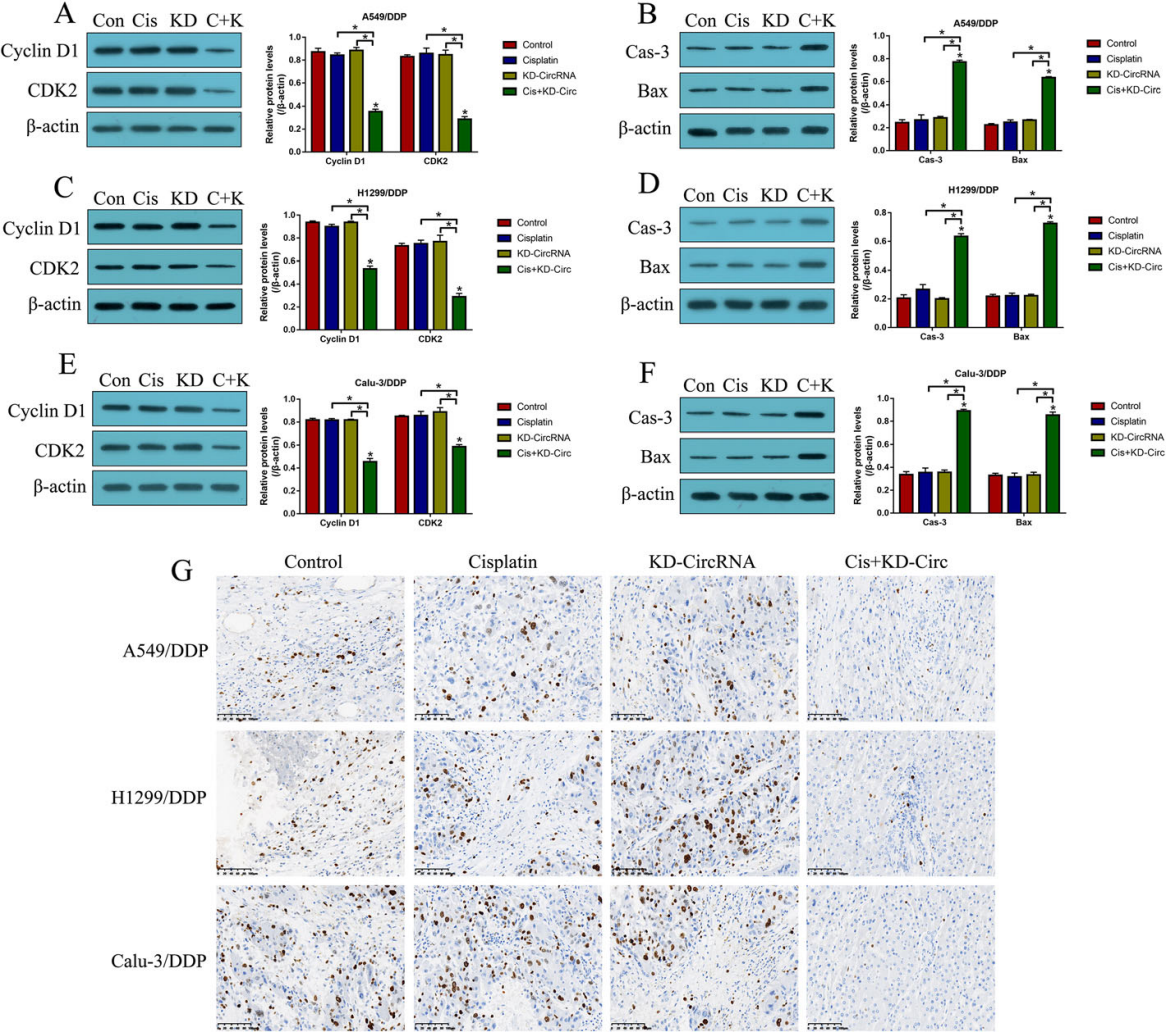
Knock-down of hsa_circRNA_103809 aggravated the inhibiting effects of cisplatin on cell growth in CR-NSCLC cells in vivo. The mice tumor tissues were collected, and Western Blot analysis was performed to examine the expression levels of Cyclin D1, CDK2, cleaved Caspase-3 and Bax in (**a, b**) A549/DDP cells (full-length blots/gels are presented in Supplementary Figure S6A-B), (**c, d**) H1299/DDP cells (full-length blots/gels are presented in Supplementary Figure S6C-D) and (**e, f**) Calu-3/DDP cells (full-length blots/gels are presented in Supplementary Figure S6E-F). **g** IHC was performed to examine the expressions and localization of Ki67 protein in mice tumor tissues, the signal intensity in different groups were assessed as follows: Control (+++), Cisplatin (+++), KD-circRNA (+++) and Cis + KD-circRNA (+). (Note: “Control: without vectors transfection and cisplatin treatment”). Each experiment was repeated at least 3 times. *P < 0.05

Next, we validated the above cellular results in vivo. To achieve this, the CR-NSCLC cells were pre-transfected with hsa_circRNA_103809 downregulation vectors, and the cells were subcutaneously injected into the dorsal flank of nude mice to establish xenograft tumor-bearing mice models. At 7 days post-injection, the tumor were subjected to high-dose cisplatin treatment every 3 days. The mice were sacrificed at day 35, and the tumor tissues were collected, prepared and analyzed by Western Blot analysis and immunohistochemistry (IHC) (Fig. 5). As shown in Fig. 5a-f, either cisplatin alone or hsa_circRNA_103809 downregulation alone had little effects on the proliferation and apoptosis associated proteins, while knock-down of hsa_circRNA_103809 and cisplatin combination treatments downregulated Cyclin D1 and CDK2 to hamper cell cycle, and upregulated cleaved Caspase-3 and Bax to trigger apoptotic cell death in A549/DDP, H1299/DDP and Calu-3/DDP cells in vivo. Consistently, the expressions and localization of Ki67 protein were examined by IHC, and the images in Fig. 5g showed that cisplatin significantly decreased the expression levels of Ki67 protein in CR-NSCLC cells with hsa_circRNA_103809 downregulation in mice tumor tissues.

## Discussion

Cisplatin is the first-line chemotherapeutic drug for non-small cell lung cancer (NSCLC) treatment in clinic [13, 14], however, long-term cisplatin treatment causes cisplatin-resistance in NSCLC cells, which seriously limits the therapeutic efficacy of this chemical drug, resulting in cancer recurrence and bad prognosis in NSCLC patients [13, 14]. Based on the above information, recent studies focused on uncovering the underlying mechanisms of cisplatin-resistance in NSCLC, and managed to identify potential therapeutic targets to improve cisplatin-sensitivity in NSCLC cells [38, 39]. Among all the cancer associated genes, researchers noticed that circular RNAs (circRNAs) were closely associated with cancer progression and drug resistance in NSCLC, and identification of novel circRNAs that regulated NSCLC pathogenesis and drug resistance became necessary and meaningful [13, 14]. Therefore, in the present study, we identified a novel circRNA, hsa_circRNA_103809, that regulated cisplatin-resistance in NSCLC. Mechanistically, according to the previous publications [36, 37], the cisplatin-resistant NSCLC (CR-NSCLC) cells were inducted from their corresponding parental cisplatin-sensitive NSCLC (CS-NSCLC) cells, and we found that hsa_circRNA_103809 tended to be highly expressed in CR-NSCLC cells, compared to CS-NSCLC cells. Interestingly, further experiments evidenced that knock-down of hsa_circRNA_103809 enhanced the inhibiting effects of cisplatin on cell proliferation and viability in CR-NSCLC cells. Furthermore, upregulation of hsa_circRNA_103809 increased cisplatin-resistance in CS-NSCLC cells, implying that targeting hsa_circRNA_103809 could potentially improve cisplatin-sensitivity in NSCLC cells. Previous data suggested that hsa_circRNA_103809 acted as an oncogene to promote cancer progression [15–19], and this study evidenced that hsa_circRNA_103809 also modulated drug resistance in NSCLC, which broadened our knowledge in this field.

Emerging evidence indicated that circRNAs sponged miRNAs to exert their biological functions [20–22], and hsa_circRNA_103809 facilitated cancer progressions by regulating miRNAs in a competing endogenous RNA (ceRNA)-dependent manner [15, 17–19, 23]. By searching the online Pubmed database (https://pubmed.ncbi.nlm.nih.gov/), we found evidence to indicate that the miRNAs, including miR-101-3p [18], miR-532-3p [15], miR-4302 [19], miR-620 [23] and miR-377-3p [17], could be sponged by hsa_circRNA_103809. Next, by performing preliminary experiments (data not shown), we surprisingly found that miR-377-3p, instead of other miRNAs, was downregulated in CR-NSCLC cells, in contrast with the parental CS-NSCLC cells, and further experiments validated the binding sites of hsa_circRNA_103809 and miR-377-3p, which were in line with the previous work [17]. Subsequently, by performing the gain- and loss-of-function experiments, we evidenced that the promoting effects of hsa_circRNA_103809 ablation on cisplatin-induced CR-NSCLC cell death were abrogated by knocking down miR-377-3p. Conversely, upregulation of miR-377-3p increased cisplatin-sensitivity in CS-NSCLC cells with hsa_circRNA_103809 overexpression, indicating that hsa_circRNA_103809 sponged miR-377-3p to regulate cisplatin-resistance in NSCLC cells, which were partly supported by the previous data [17].

Glutamate oxaloacetate transaminase 1 (GOT1) is crucial for promoting cancer progression by regulating glutamate metabolism [31, 32], and inhibition and silencing of GOT1 had been validated as an effective strategy to impair cancer growth [31, 32]. Interestingly, previous data suggested that GOT1 could be regulated by cisplatin [33], and our experiments validated that hsa_circRNA_103809 positively regulated GOT1 in NSCLC cells through miR-377-3p. Mechanistically, there existed binding sites between miR-377-3p and 3’UTR of GOT1 mRNA, and miR-377-3p negatively regulated GOT1 in NSCLC cells at both transcriptional and translational levels. In addition, we noticed that upregulation of hsa_circRNA_103809 increased GOT1 expression levels, which were reversed by overexpressing miR-377-3p, implying that hsa_circRNA_103809 sponged miR-377-3p to upregulate GOT1 in NSCLC cells. Furthermore, knock-down of hsa_circRNA_103809 increased cisplatin-sensitivity in CR-NSCLC cells, while overexpression of hsa_circRNA_103809 increased cisplatin-resistance in CS-NSCLC cells, which were reversed by overexpressing and silencing GOT1, suggesting that hsa_circRNA_103809 upregulated GOT1 to modulate cisplatin-resistance in NSCLC cells. Finally, by performing the in vivo experiments, we evidenced that knock-down of hsa_circRNA_103809 triggered apoptotic cell death to inhibit tumorigenesis in the xenograft tumor-bearing mice models.

Interestingly, recent data noticed that NRF2 mediated glutamine metabolism was closely associated with chemo-resistance in pancreatic cancers [40], given that GOT1 served as a crucial regulator for glutamate metabolism, we hypothesized that there might exist connections between NRF2 and GOT1 in regulating cisplatin-resistance in NSCLC. However, the detailed mechanisms are still needed to be studied. In addition, since Kirsten rat sarcoma viral oncogene homolog (KRAS) is one of the driver gene of NSCLC [41], it was worthy to investigate the interplay between KRAS gene and the hsa_circRNA_103809/miR-377-3p/GOT1 pathway in regulating NSCLC development in our future work.

## Conclusions

Taken together, through in vitro and in vivo experiments, this study found that targeting the hsa_circRNA_103809/miR-377-3p/GOT1 pathway inhibited cell proliferation and viability, and triggered cell apoptosis to increase cisplatin-sensitivity in NSCLC cells. Our work broadened our knowledge in this filed, and provided potential therapeutic targets to improve the therapeutic efficacy of current chemical drug for NSCLC.

## Supplementary Information

**Additional file 1:** **Figure S1.** The overexpression and downregulation vectors for (A) hsa_circRNA_103809, (B) miR-377-3p, and (C) GOT1 overexpression vectors were delivered into cisplatin-resistant A549/DDP, H1299/DDP and Calu-3/DDP cells, respectively, and examined by using the Real-Time qPCR analysis. (Note: “Control: without vectors transfection”). Each experiment was repeated at least 3 times. *P < 0.05.

**Additional file 2:** **Figure S2.** The overexpression and downregulation vectors for (A) hsa_circRNA_103809, (B) miR-377-3p, and (C) GOT1 downregulation vectors were delivered into cisplatin-sensitive A549, H1299 and Calu-3 cells, respectively, and examined by using the Real-Time qPCR analysis. (Note: “Control: without vectors transfection”). Each experiment was repeated at least 3 times. *P < 0.05.

**Additional file 3:** **Figure S3.** The schematic image for luciferase vectors.

**Additional file 4:** **Figure S4.** The uncropped full-length gels and blots for Fig. 1k in A549 cells, H1299 cells and Calu-3 cells, respectively. “#1”, “#2“ and ”#3″ indicated three times of repetition for GOT1 protein, and each lane was labelled according to the cropped gels/blots in Fig. 1k.

**Additional file 5:** **Figure S5.** The uncropped full-length gels and blots for (A) Fig. 2l, (B) Fig. 2n and (C) Fig. 2p in A549 cells, H1299 cells and Calu-3 cells, respectively. “#1”, “#2“ and ”#3″ indicated repetitions for each experiment, and each lane was labelled according to the cropped gels/blots in Fig. 2, Fig. 2n and Fig. 2p.

**Additional file 6:** **Figure S6.** The uncropped full-length gels and blots for (A) Fig. 5a, (B) Fig. 5b, (C) Fig. 5c, (D) Fig. 5d, (E) Fig. 5e and (F) Fig. 5f. and each lane was labelled according to the cropped gels/blots in Fig. 5a-f.

## Data Availability

All the data and materials involved in this study had been included in the manuscript.

## Abbreviations

NSCLC: Non-small cell lung cancer
circRNAs: Circular RNAs
CS-NSCLC: Cisplatin-sensitive NSCLC
CR-NSCLC: Cisplatin-resistant NSCLC
GOT1: Glutamate oxaloacetate transaminase 1
Glu: Glutamate
3’UTRs: 3′ untranslated regions
ceRNA: Competing endogenous RNA
IHC: Immunohistochemistry
