# Supplementary figures and images for: A novel circular RNA hsa_circRNA_103809/miR-377-3p/GOT1 pathway regulates cisplatin-resistance in non-small cell lung cancer (NSCLC)
Source: BMC Cancer. 2020 Dec 4;20:1190. doi: 10.1186/s12885-020-07680-w (PMC7716498; doi:10.1186/s12885-020-07680-w)

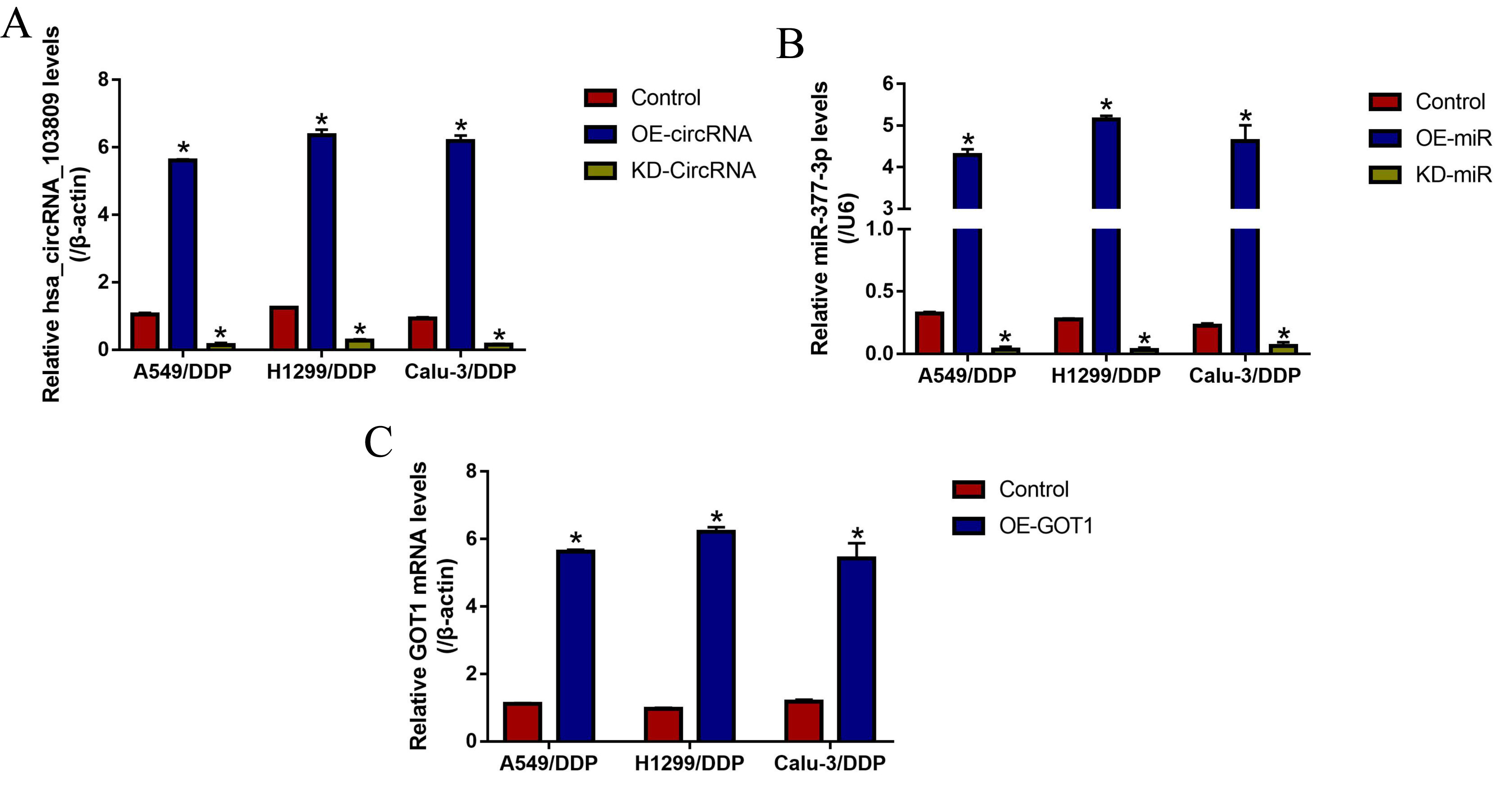

Supplement: Supplementary file 1 — Additional file 1: Figure S1. The overexpression and downregulation vectors for (A) hsa_circRNA_103809, (B) miR-377-3p, and (C) GOT1 overexpression vectors were delivered into cisplatin-resistant A549/DDP, H1299/DDP and Calu-3/DDP cells, respectively, and examined by using the Real-Time qPCR analysis. (Note: “Control: without vectors transfection”). Each experiment was repeated at least 3 times. *P < 0.05. [file 12885_2020_7680_MOESM1_ESM.jpg]

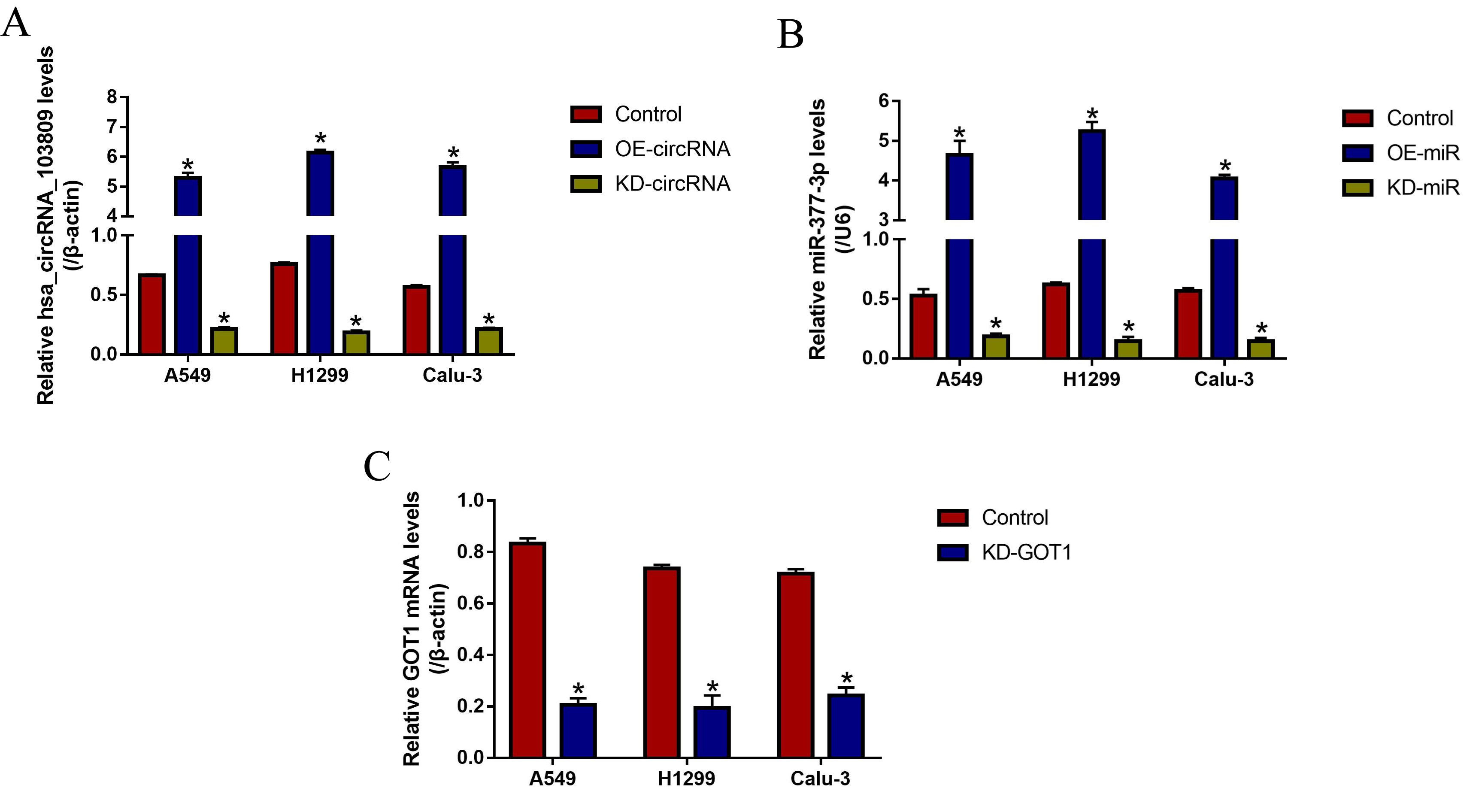

Supplement: Supplementary file 2 — Additional file 2: Figure S2. The overexpression and downregulation vectors for (A) hsa_circRNA_103809, (B) miR-377-3p, and (C) GOT1 downregulation vectors were delivered into cisplatin-sensitive A549, H1299 and Calu-3 cells, respectively, and examined by using the Real-Time qPCR analysis. (Note: “Control: without vectors transfection”). Each experiment was repeated at least 3 times. *P < 0.05. [file 12885_2020_7680_MOESM2_ESM.jpg]

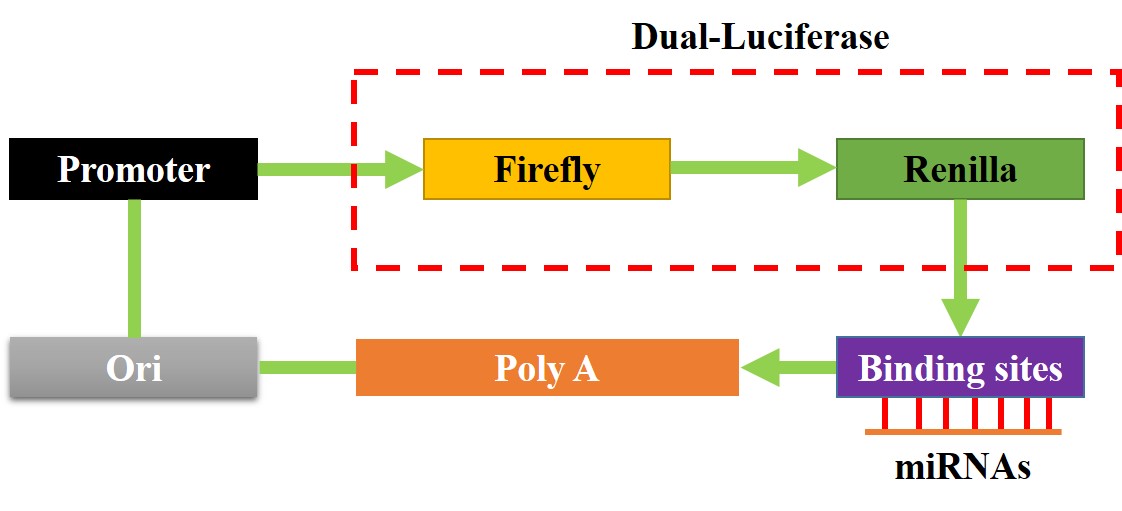

Supplement: Supplementary file 3 — Additional file 3: Figure S3. The schematic image for luciferase vectors. [file 12885_2020_7680_MOESM3_ESM.jpg]

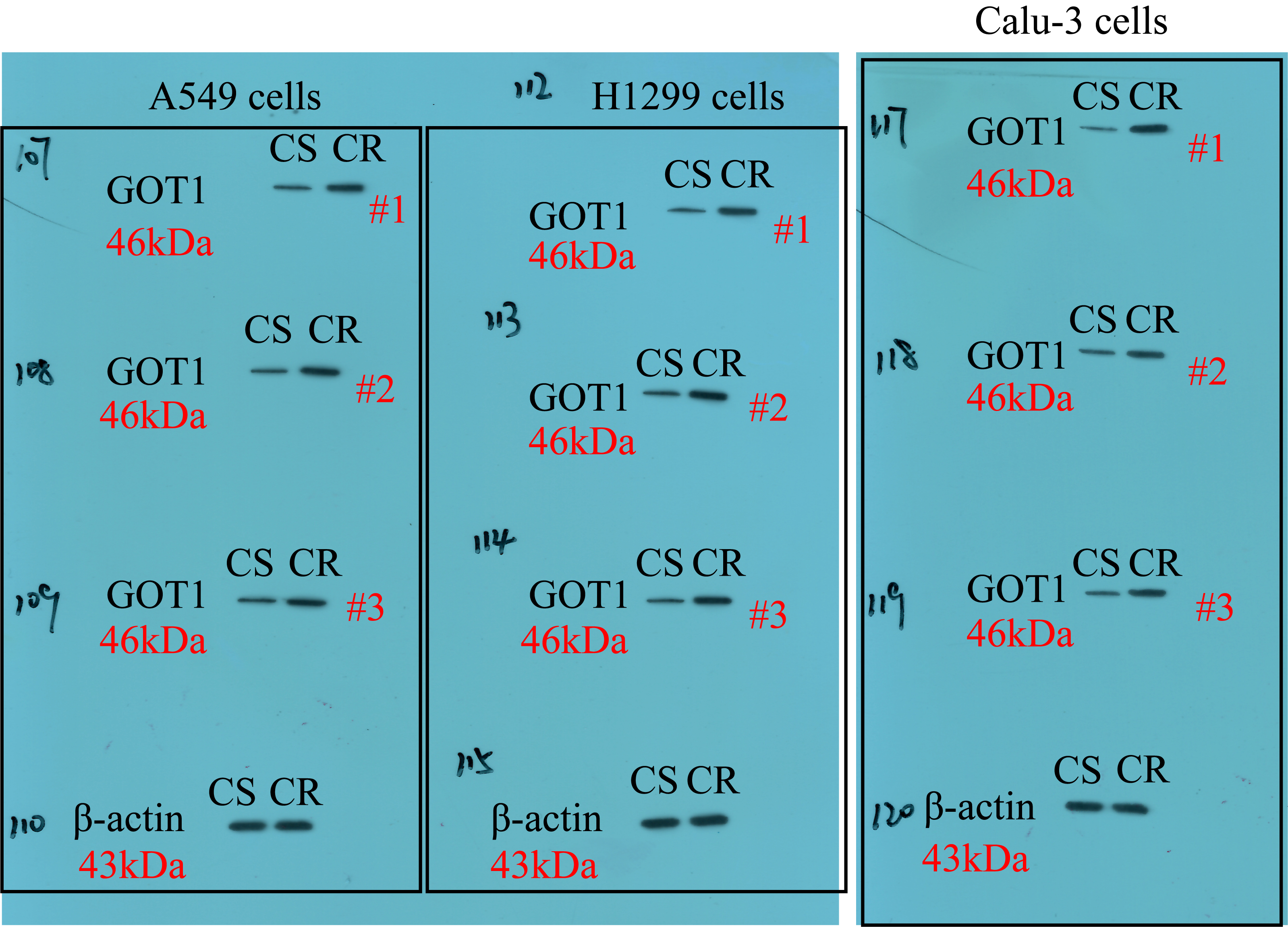

Supplement: Supplementary file 4 — Additional file 4: Figure S4. The uncropped full-length gels and blots for Fig. 1k in A549 cells, H1299 cells and Calu-3 cells, respectively. “#1”, “#2“ and ”#3″ indicated three times of repetition for GOT1 protein, and each lane was labelled according to the cropped gels/blots in Fig. 1k. [file 12885_2020_7680_MOESM4_ESM.jpg]

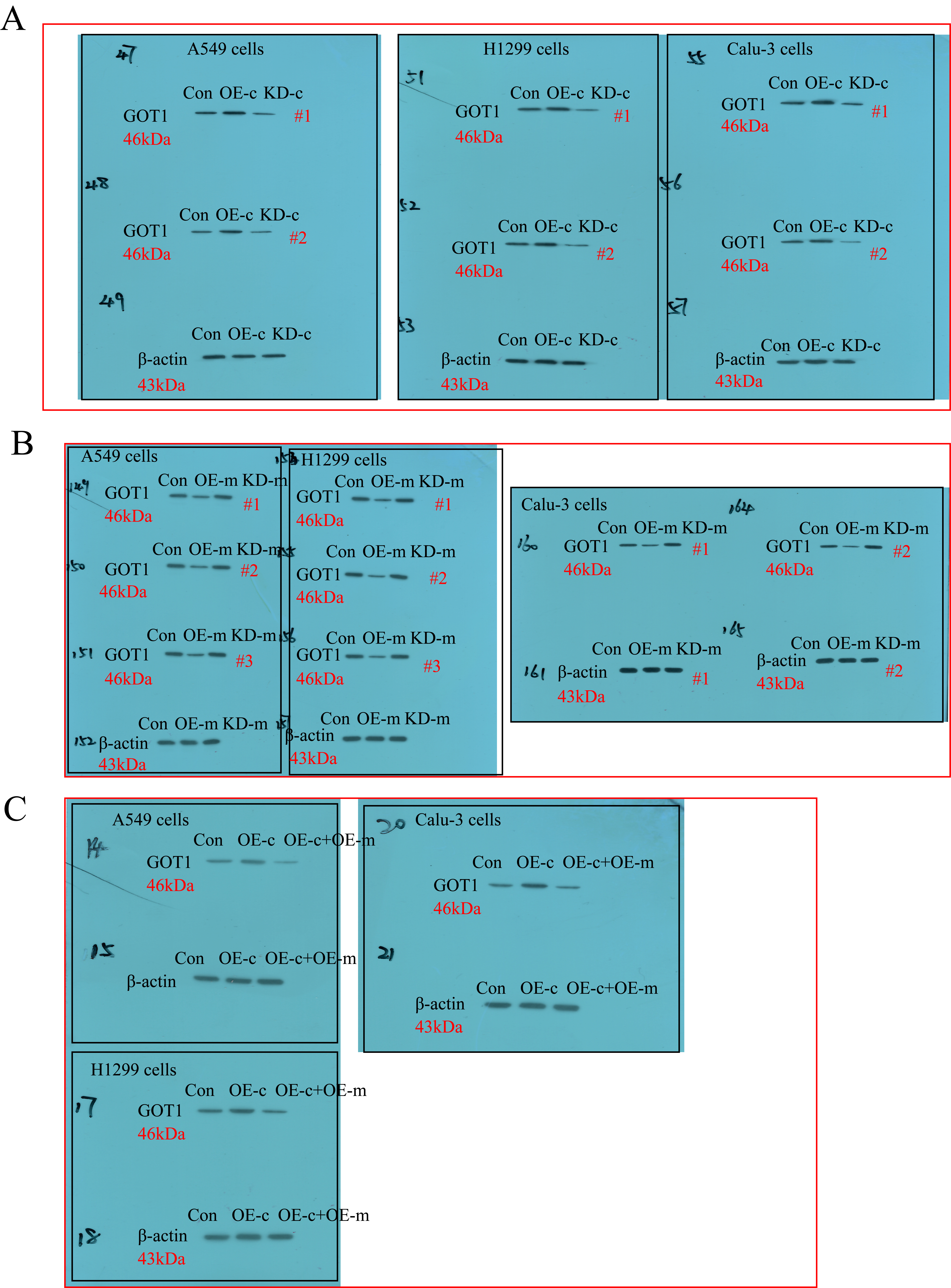

Supplement: Supplementary file 5 — Additional file 5: Figure S5. The uncropped full-length gels and blots for (A) Fig. 2l, (B) Fig. 2n and (C) Fig. 2p in A549 cells, H1299 cells and Calu-3 cells, respectively. “#1”, “#2“ and ”#3″ indicated repetitions for each experiment, and each lane was labelled according to the cropped gels/blots in Fig. 2, Fig. 2n and Fig. 2p. [file 12885_2020_7680_MOESM5_ESM.jpg]

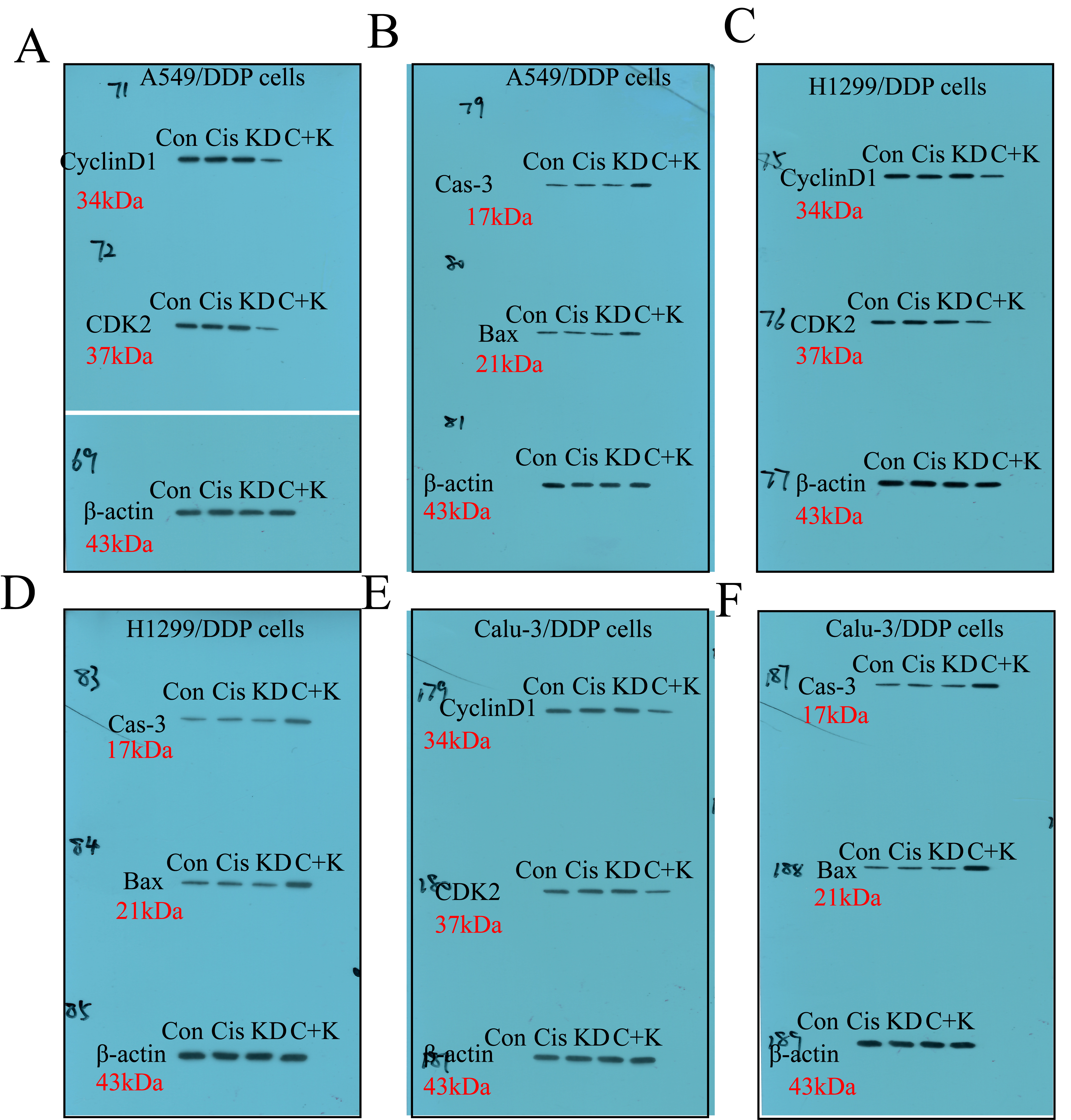

Supplement: Supplementary file 6 — Additional file 6: Figure S6. The uncropped full-length gels and blots for (A) Fig. 5a, (B) Fig. 5b, (C) Fig. 5c, (D) Fig. 5d, (E) Fig. 5e and (F) Fig. 5f. and each lane was labelled according to the cropped gels/blots in Fig. 5a-f. [file 12885_2020_7680_MOESM6_ESM.jpg]
